# Spatiotemporal patterns and influencing factors of coupling coordination development of ecosystem services and urbanization: a case of western border counties in China

**DOI:** 10.3389/fpubh.2025.1579775

**Published:** 2025-04-22

**Authors:** Luyao Liu, Yungui Shi, Qun Meng

**Affiliations:** ^1^School of Public Administration, Green Development and Borderland Governance Research Institute, Guangxi University, Nanning, China; ^2^Zhejiang University of Finance and Economics Department, School of Public Administration, Hangzhou, China

**Keywords:** border counties, ecosystem service value, urbanization, coupling coordination, influencing factor

## Abstract

**Introduction:**

The spatial distribution of border regions highly overlaps with key ecological function zones and economically underdeveloped areas, making the coordination between urbanization and ecosystem service value (ESV) crucial.

**Methods:**

Taking China's western border counties as a case study, this study explores the spatiotemporal variation characteristics of ESV, urbanization levels, and their coupling coordination relationship. By applying the Obstacle Degree Model and the geodetector analysis, we identify the factors that obstruct and drive the coordinated development of the ESV-urbanization coupling system from both internal and external perspectives.

**Results:**

(1) From 2000 to 2020, ESV in China's western border counties declined gradually, while the urbanization increased with significant spatial imbalances in development. (2) In most border counties, the coupling coordination degree (CCD) of ESV and urbanization was either mildly or severely unbalanced, and a growing number of counties experienced lagging ESV. In particular, some counties exhibited synchronous declines in both ESV and urbanization, posing a significant threat to regional sustainable development. (3) The main obstacle factors of CCD include social security, industrial structure and ecological regulation functions, while the primary driving factors are precipitation, temperature, and net primary productivity (NPP). (4) The interaction of tourism, stable ecological areas and government actions with other factors significantly enhances the driving effect on CCD in border counties.

**Conclusion:**

This study provides policy recommendations and a practical basis for promoting synergistic development between ESV and urbanization in China's western border counties and other similar border regions.

## 1 Introduction

Ecosystem services (ES) provide critical benefits to human societies through direct or indirect contributions from natural ecosystems (1). These services compass provisioning (e.g., food production), regulating (climate modulation), and cultural functions (e.g., aesthetic landscapes), forming essential linkages between ecological and socioeconomic systems (2). Since the advent of the Anthropocene, the global urbanization has precipitated severe ecological crises, including resource depletion, environmental contamination, biodiversity loss, extreme weather events (3). These pressures have triggered widespread ES degradation, profoundly threatening human well-being. At present, the world is facing great pressure of ecosystem protection. Meanwhile, urbanization remains a vital engine for economic advancement in developing countries (4). The inherent conflict with ES preservation demands urgent resolution through targeted interventions (5, 6).

The regulation of the ES-urbanization nexus has emerged as a critical global challenge, engaging nations worldwide. In response, international scientific initiatives—including the Future Earth (FE), the IPCC Program, the 2030 Sustainable Development Goals (SDGs), and Coupled Human and Natural Systems (CHANS)—have been established to synergize socioeconomic development with ecosystem protection, ultimately advancing regional sustainability and human wellbeing.

The ES-urbanization relationship constitutes a coupled development system characterized by mutual influence, coordination and synergies (7). Academic inquiry into this coupling mechanism commenced early, with scholars systematically exploring theoretical frameworks, modeling approaches and impacting factors to establish foundational knowledge (8). Notable conceptual advancements include the “telecoupling” (9), “metacoupling” (10), and “Coupled Human and Natural Cube” (11) which elucidate complex interaction mechanisms. Methodologically, Ecosystem service value (ESV) has emerged as the predominant metric for ES quantification due to its operational tractability and monetary comparability (12), while urbanization assessments typically integrate economic, demographic, and spatial urbanization (13–15). In addition, with the development of remote sensing technology, multi-period remote sensing data has further support the quantitative estimation of the ES, the urbanization, and the coupling relationship between the two (16, 17).

Generally speaking, current research on the ES-urbanization nexus exhibits three limitations. First, analytical approaches focus on isolated dimensions-examining either specific ES components [e.g., provisioning service (18)], or singular urbanization aspects [e.g., urban population ratio (19) or urban land expansion (20)], while neglecting their systemic interactions (21). Moreover, most scholars have employed correlation analysis (21) and econometric regression models (22, 23) to examine the relationship between the two. Few studies have utilized the coupling coordination degree model to explore their non-linear interactions, with most research focusing solely on analyzing the spatiotemporal variation characteristics of coupling coordination degree (24, 25). Second, in terms of determinant analysis, linear regression (26), Granger causality test (27) and geographic detectors (28, 29) were used to examine the key drivers. Notably, ES and urbanization constitute a complex coupled system, but few scholars analyze its impact factors from the perspective of internal and external systems. Third, as to the global terrestrial ecosystems, scholarly attention remains disproportionately concentrated on inland river basins (30), cities (31, 32) and urban agglomerations (33–35), instead of the border regions.

Border regions predominantly occupy socioeconomically marginalized areas, and ecologically fragile areas, exhibiting significant spatial overlap with critical ecological function areas (36, 37). These regions commonly confront the dual imperative of urbanization advancement and ecological preservation. Southeast Asia borderlands exemplify this challenge, where deregulated environmental regulations have been made to promote urbanization, aiming to address the prominent issue of socio-economic inequalities, improve the livelihoods of local populations, and stimulate the development of border economies (38, 39). However, such practices have led to an increased risk of ecological degradation. The coupling coordinated development of ESV and urbanization is required to ensure the sustainable development of the border region.

China's border regions characterized economically underdeveloped and ecologically fragile environments. Abundant in diverse ecosystems and energy resources, China's western borderlands provide various types of ES. To prevent the degradation of their ecological functions and the reduction of ES, most of China's western border regions have been designated as national key ecological function areas with stringent ecological protection measures. Concurrently, China has released many national policies to promote the urbanization of these border regions (40), while recent high-quality development policies emphasize the balanced development between ecological preservation and urban growth. However, China's western border counties regions still exhibit persistent developmental disparities compared to inland regions (41). This context underscores an urgent need to resolve the complex challenge of the coupling coordinated development between ecosystem preservation and urban growth, but the main factors impacting on this coordination remain insufficiently understood. Notably, the county scale is the fundamental administrative unit for the policy implementation, which is also an important level for precise control of territorial spatial functions and promotion of urbanization development in China (29, 42, 43).

This study selected China's western border counties (2000–2020) through five sequential analyses: (1) Framework development for coupling ESV and urbanization in western border counties. (2) Composite evaluation of ESV and urbanization level from 2000 to 2020. (3) CCD assessment and the development relationship of the two systems. (4) Identification of the obstacles and driving factors impacting on the CCD. (5) Formulation of the relevant policy recommendations for border counties to achieve coupling coordinated development.

Aiming to establish an evidence-based foundation for the synergistic growth of ESV-urbanization development in border counties, this study contributes in two key areas. (1) Conceptual framework of coupling development: construction of a coupled ESV and urbanization theoretical model that expands disciplinary boundaries. (2) Analytical advancement: A dual-system analysis was conducted to identify key determinants factors impacting ESV and urbanization synergies from both internal and external perspectives of the system based on obstacle factors and driving factors.

## 2 Data source and methods

### 2.1 Theoretical framework

Urbanization can be represented in a series of complex evolution and transformation processes encompassing the growth of urban population, expansion of urban space, and industrial transformation, which is an inevitable trajectory of regional socioeconomic development (44). Based on the basic attributes of ecosystems across spatiotemporal scales, the ES deliver critical benefits including food production, climate regulation, environmental purification, aesthetic landscape. These services are systematically categorized into four types: Provisioning, regulating, supporting and cultural services (1, 45). Urbanization inherently induces ecological land conversion, disrupts ecosystem structures, and alters the ES and ESV, ultimately impacting the sustainable development and human wellbeing of the region.

**Figure 1:**
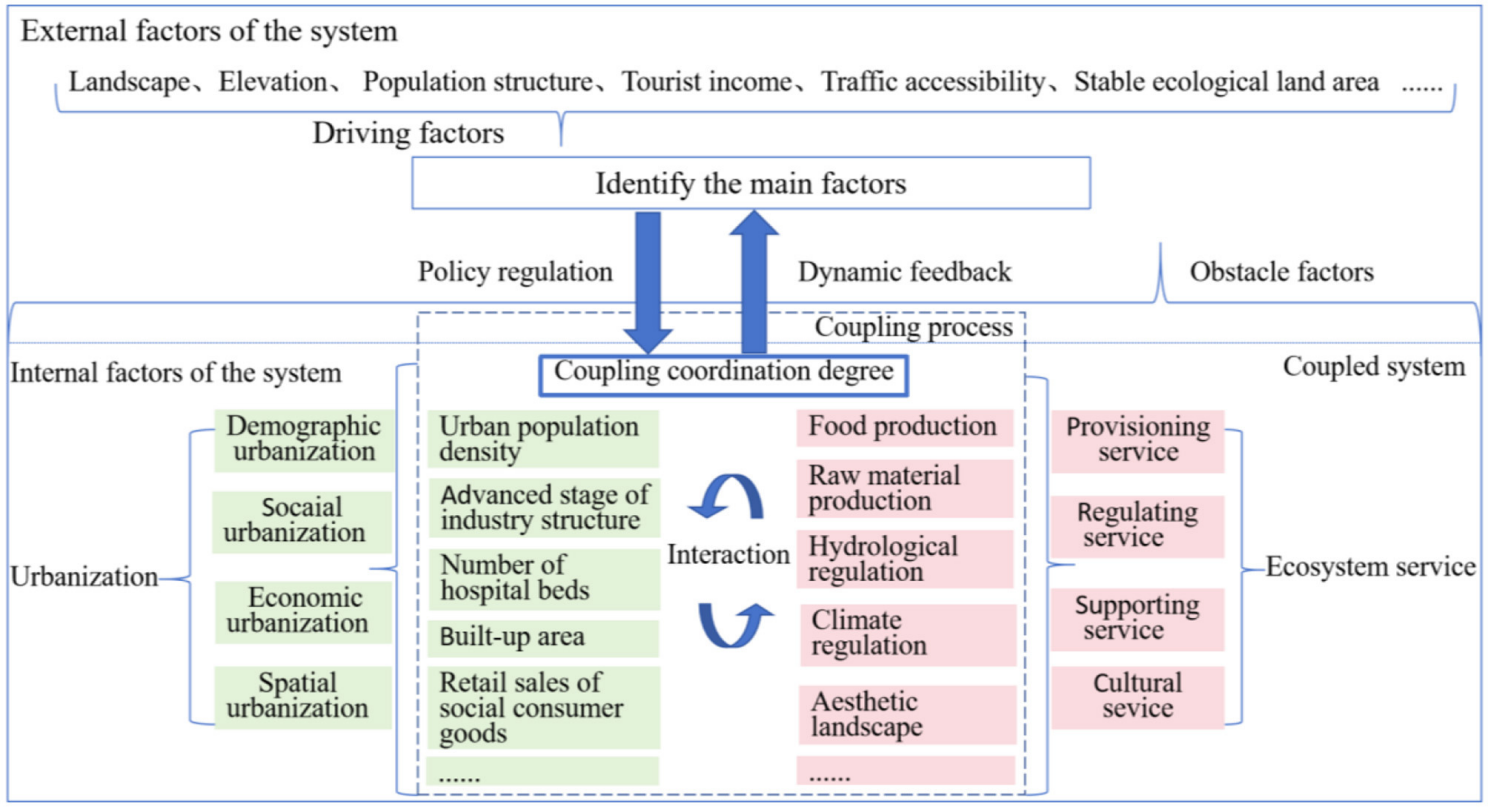
Theoretical framework of the balance between ES and urbanization.

The concept “Coupling”-originated from physics-is used to describe the interdependent interactions between two or more systems (46). Both ES and urbanization are complex dynamic systems: the former mainly includes provisioning, regulating, supporting and cultural service subsystem while the latter mainly includes demographic, social, economic and land-use urbanization subsystem. From the perspective of comprehensive system theory and coupling principles (Figure 1), ES and urbanization exhibit mutual promotion and constraint, forming an open, multi-element, multi-level coupled system. Their interactions are mediated by natural, socioeconomic, and governance factors.

From the view of coupling process, the pace, scale, and level of urbanization development will influence quantitative and qualitative changes in ecosystem services. Conversely, improvement or degradation of ecosystem services correspondingly accelerates or constrains urbanization. As urbanization progresses into the middle-late stages of high-quality development, its positive feedback on ecosystem health will be significantly enhanced. This evolution will consequently surpass the turning point of the Environmental Kuznets Curve (47), transitioning the coupling system of the ESV and urbanization into a phase of coupled coordinated development. Unlike developed inland regions, border areas are profoundly shaped by geopolitical forces, exhibiting unique urbanization trajectories. Concurrently, border ecosystems demonstrate heightened sensitivity, with ecological changes characterized by inherent uncertainty. The urbanization and ecosystem services of border counties engage in complex interactions driven by factors such as cross-border population flows, international engagement, and specialized policies. Revealing the coupling relationship between ES and urbanization and its temporal-spatial variation law, analyzing the main impact factors of their coupling coordinated development and implementing targeted interventions can advance regional sustainable development.

### 2.2 Study area

**Figure 2:**
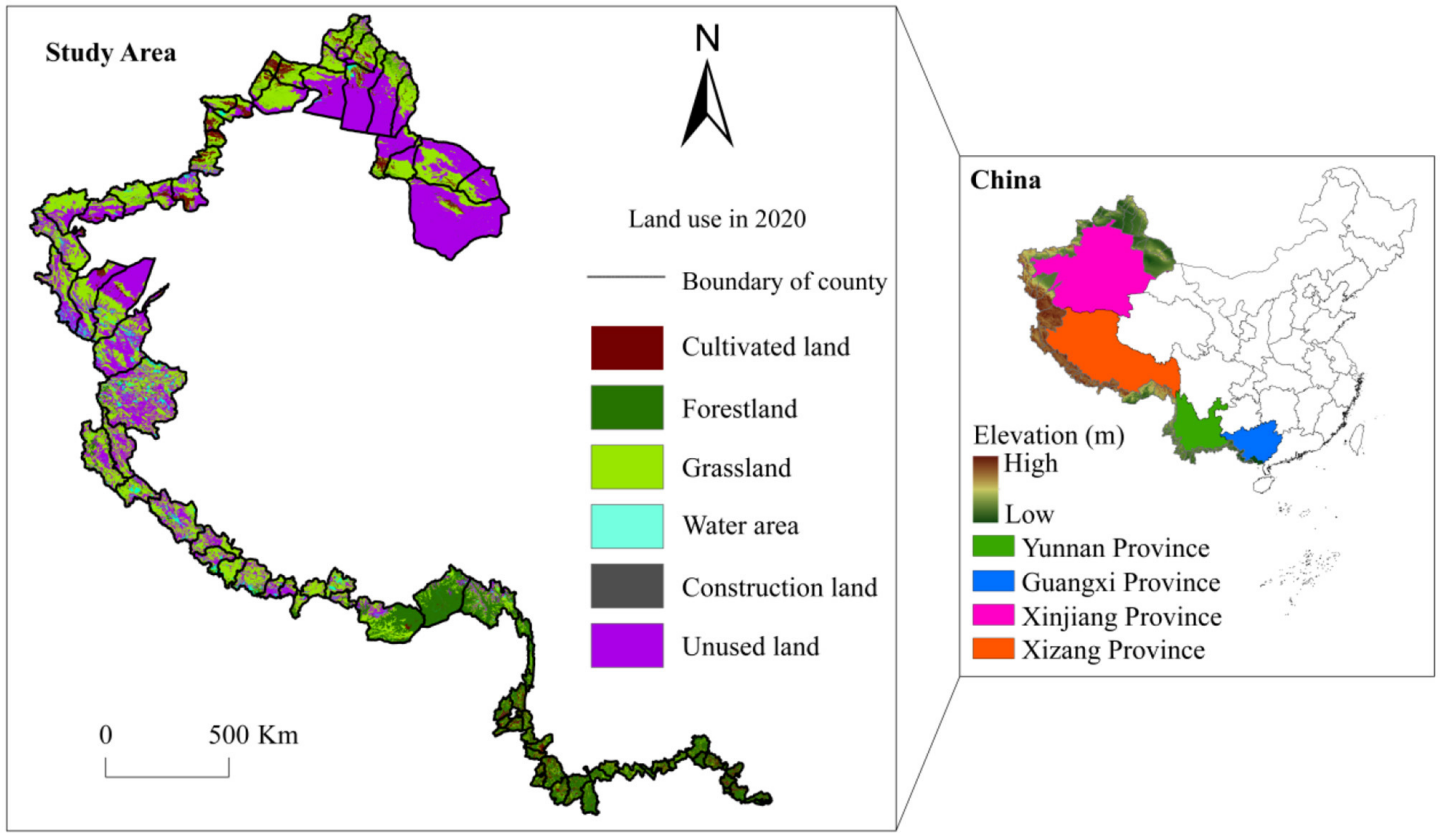
Location of the study area.

China's western border is the borderline accessing China's western land and consists of the border counties under the jurisdiction of Xinjiang, Xizang, Guangxi and Yunnan Province (73°26′E−109°47′E, 21°08′N−49°11′N). To ensure consistency of the research units over the long time series, the county units (including counties, county-level cities, and municipal districts) with administrative division changes during the research period and their corresponding statistical data were corrected. Finally, a total of 83 basic research units were acquired (Figure 2). The study areas border 14 countries, including Kazakhstan, Afghanistan, India, Nepal, and Myanmar, and represent the region with the most land neighbors and the most extensive border in China. This border stretches over 14,680 km and comprises 66.73% of the nation's total land border. As of 2020, 94% of the border counties in the study area had a per capita GDP below the national average of 71,830 yuan per person. Urbanization is needed to drive economic growth to reduce regional disparities. The local lands were used in diverse ways, mainly including unused land, grassland and forestland, which account for 40.10%, 36.34%, and 14.87%, respectively. The soil in most areas is barren, ecologically fragile and vulnerable to urbanization. The development of urbanization will further lead to the degradation and even disappearance of ecological land in the study area, posing a serious threat to regional sustainable development. In addition, some counties are located in cold, high-elevation and desert arid areas, which are not conducive to population aggregation. At the same time, the special topographies, such as plateau and mountain, are not conducive to urban construction and spatial expansion, further restricting the development of urbanization. In summary, the contradiction between ecosystem protection and urbanization development in the western border counties of China is prominent. Promoting the coupling coordinated development of the two is essential for the sustainable development of these border counties.

### 2.3 Data source

The county-scale panel data on China's western border regions from 2000 to 2020 were collected in this study from the land use interpretation data for 2000, 2005, 2010, 2015, and 2020, provided by the Scientific Data Center of Resources and Environment Science of Chinese Academy of Sciences (https://www.resdc.cn). These data mainly include information on land use, nature, society and economy. The spatial resolution is 30 meters, and the accuracy is above 90%. The land use types were reclassified into 6 first-level categories: urban and rural construction land, unused land, water area, grassland, forestland and cultivated land. For natural data, net primary productivity (NPP), annual average temperature, and annual precipitation data were sourced from the National Platform for Basic Conditions of Science and Technology-National Earth System Science Data Center (http://www.geodata.cn). Data on soil organic carbon were obtained from the Harmonized World Soil Database (HWSD), and data on soil conservation were from the Scientific Data Bank (SDB) (48). The socioeconomic data were primarily derived from *Xinjiang Statistical Yearbooks* (2000-2021), *Tibet Statistical Yearbooks* (2000–2021), *Yunnan Statistical Yearbooks* (2000–2021), *Guangxi Statistical Yearbooks* (2000–2021), *China County Statistical Yearbooks* (2000–2021), *National Agricultural Product Cost Price Compilation* and *China Statistical Yearbooks* (2000–2021). Several missing data points in individual years were supplemented using multiple linear interpolation method.

### 2.4 Methods

#### 2.4.1 Quantification of ESV

ESV acts as an important measure of ES function and has been widely recognized and applied in academia (49, 50). This study is carried out based on the unit area ecological service value equivalent table of Chinese ecosystem, which was duly established by Gaodi Xie according to China's realities and the rule that the ESV of a standard equivalent factor is equal to 1/7 of the average grain yield market value of the study area that year (51, 52). To eliminate the influence of currency fluctuation, the ESV of a multi-year standard equivalent factor in the study area was 1,481.48 yuan/hm^2^, which was calculated based on the per unit area yield of grain and grain unit price of China's western border counties from 2000 to 2020. Considering that ecosystem functions including food production, raw material production, gas regulation, climate regulation, environmental purification, conservation nutrient cycling biodiversity, and aesthetic landscape services are positively correlated with biomass; water supply and hydrological regulation functions are related to precipitation variations; and soil conservation is predominantly influenced by rainfall, terrain relief, soil properties, and vegetation coverage. NPP, precipitation, and soil conservation were selected as adjustment factors to correct for the spatiotemporal differences in ESV (51). The specific formula can be expressed as (21, 53):


(1) $$\begin{array}{l} ESV=D\times \sum_{1}^{n}A_{i}\times F_{nij} \end{array}$$

(2) $$F_{nij}=\{\begin{array}{c} P_{ij}\times F_{n1}\;or\\ R_{ij}\times F_{n2}\;or\\ S_{ij}\times F_{n3}\; \end{array}\;$$

(3) $$\begin{array}{l} P_{ij}=\frac{B_{ij}}{\bar{B}} \end{array}$$

(4) $$\begin{array}{l} R_{ij}=\frac{W_{ij}}{\bar{W}} \end{array}$$

(5) $$\begin{array}{l} S_{ij}=\frac{E_{ij}}{\bar{E}} \end{array}$$

Where, *ESV* is ecosystem service value, *D* is *ESV* of a standard equivalent factor, *n* is the type of ecosystem service, *i* is land use type, *j* is the year, *A*_*i*_ is the land area, *F*_*n*1_, *F*_*n*2_, and *F*_*n*3_ are the service functions related to NPP, precipitation, and soil conservation, respectively; *P*_*ij*_, *R*_*ij*_, and *S*_*ij*_ are the NPP, precipitation and soil conservation spatiotemporal adjustment factors, respectively; *B*_*ij*_, *W*_*ij*_, and *E*_*ij*_ are the NPP, precipitation, and soil conservation amounts per unit area of the *i-*th land use type in the *j-*th year, respectively; $\bar{B}$, $\bar{W}$, and Ē are the NPP, precipitation and soil conservation amounts per unit area of the whole country, respectively. Stable ESV is the total ESV provided by land with no change in land use type during the study period.

#### 2.4.2 Horizontal measurement of urbanization

According to the realities of the study area and relevant literatures (13, 26, 33, 54), 11 indicators were selected from four aspects of demographic urbanization, social urbanization, economic urbanization, and spatial urbanization to construct the urbanization evaluation indicator system of China's western border counties (Table 1). In the comprehensive evaluation of urbanization level, the data were standardized by the range method to eliminate the impact of different dimensions and orders of magnitude on the evaluation indicators (13). Then, the entropy method was used to calculate the weight of each indicator (55). Finally, the urbanization level was evaluated using the linear weighting method. The specific method can be expressed as (16):


(6) $$\begin{array}{l} K=\sum_{1}^{n}T_{i}Z_{i} \end{array}$$

Where, *K* is the urbanization level indicator, *n* is the number of indicators, *T*_*i*_ is the weight of indicators of the *i-th* indicator, and *Z*_*i*_ is the standardized value of the *i-th* indicators. In order to verify the robustness of the results, sensitivity analyses have been conducted on ESV and urbanization level, respectively (56).

**Table 1:** Index system for measuring the urbanization level in border counties.

**Subsystem**	**Serial code**	**Indicator layer**	**Unit**	**Indicator description**	**Indicator attribute**	**Weight**
Demographic urbanization	X_1_	Proportion of urban population to total population	%	Reflects regional demographic changes	+	0.02
	X_2_	Urban population density	Person/m^2^	Reflects the concentration of urban population	+	0.05
Economic urbanization	X_3_	Per capita GDP	Yuan	Reflects the level of regional economic development	+	0.04
	X_4_	Advanced stage of industry structure	%	Reflects the adjustment of regional industrial structure Tertiary-to-secondary industry ratio reflecting economic structural optimization	+	0.37
	X_5_	Per capita fixed assets investment	10^4^ yuan/person	Reflects the investment in township development	+	0.08
	X_6_	Outstanding amount of savings deposit of urban and rural residents	10^4^ yuan	Reflects the per capita living standards	+	0.08
Social urbanization	X_7_	Number of hospital beds	Beds/person	Reflects the level of medical resources protection	+	0.11
	X_8_	Number of middle school students per 10,000 people	Person	Reflects the progress of population quality and the scale of education development	+	0.04
	X_9_	Retail sales of social consumer goods	10^4^ Yuan	Reflects the regional social consumption level	+	0.07
Spatial urbanization	X_10_	Built-up area	km^2^	Reflects the scale of urban construction land	+	0.06
	X_11_	Land development intensity index	%	Reflects the intensity of regional land development	+	0.08

#### 2.4.3 Coupled coordination models

Coupled coordination models can intuitively reflect the coupling state achieved through the interaction of two systems under the influence of internal and external factors, allowing for the quantification of the strength of their coupling state (54). The formulas are as follows (57):


(7) $$\begin{array}{l} C=\frac{2\sqrt{E\times K}}{\left(E+K\right)} \end{array}$$

(8) $$\begin{array}{l} T=\alpha E+\beta K \end{array}$$

(9) $$\begin{array}{l} CCD=\sqrt{C\times T} \end{array}$$

Where, *C* is the coupling degree, *E* and *K* are the comprehensive evaluation values of ESV and urbanization, respectively; *T* is the comprehensive coordination indicator, α and β are the weight coefficients of the two systems, and their relative sizes basically do not affect the overall trend of *CCD* in the model. Equal importance is applied to the ES and urbanization in this paper, and both are given the value of 0.5 (58). *CCD* is the coupling coordination degree; the greater the *CCD* value, the higher the degree of coupling coordination. By referring to previous studies (54, 59), the CCD were divided into five classifications and 15 sub-classifications as shown in Table 2.

**Table 2:** Classification criterion of CCD.

**Classification**	**Range**	**Systematic comparison**	**Sub-classification**
Highly balanced	(0.8, 1]	E – K > 0.1	Urbanization lag
		0 ≤ | E– K | ≤ 0.1	Systematically balanced
		K – E > 0.1	ESV lag
Moderately balanced	(0.6, 0.8]	E – K > 0.1	Urbanization lag
		0 ≤ | E – K | ≤ 0.1	Systematically balanced
		K – E > 0.1	ESV lag
Basically balanced	(0.4, 0.6]	E – K > 0.1	Urbanization lag
		0 ≤ | E – K | ≤ 0.1	Systematically balanced
		K – E > 0.1	ESV lag
Moderately unbalanced	(0.2, 0.4]	E – K > 0.1	Urbanization lag
		0 ≤ | E– K | ≤ 0.1	Systematically balanced
		K – E > 0.1	ESV lag
Seriously unbalanced	(0, 0.2]	E – K > 0.1	Urbanization lag
		0 ≤ | E – K | ≤ 0.1	Systematically balanced
		K – E > 0.1	ESV lag

#### 2.4.4 Obstacle degree model

The obstacle degree model enables systematic of constraining factors and their relative impediment magnitudes within the coupled system of ES and urbanization. The specific formula can be expressed as (60):


(10) $$\begin{array}{l} F_{ij}=1-U_{ij} \end{array}$$

(11) $$\begin{array}{l} Y_{ij}=\frac{W_{ij}F_{ij}}{\sum_{j=1}^{m}W_{ij}F_{ij}}\times 100\% \end{array}$$

Where *F*_*ij*_ is the deviation degree of the indicator *i* in the *j-*th year, indicating the gap between the indicator *i* and *CCD*; *U*_*ij*_ is the standardized value of indicator *i* in year *j*; *w*_*ij*_ represents the weight of indicator; *Y*_*ij*_ denotes the obstacle degree of the indicator *i* to *CCD*.

#### 2.4.5 Model of geographic detector

Based on established methodologies (52, 61) and considering the realities of the study area, 16 indicators across natural, economic and social dimensions were selected to analyze the primary external driving factors of the coupled system (Table 3). The factor detection module in the geographic detector was used to calculate the explanatory power of individual factor on the driving effect of coupling development of urbanization and ES. Additionally, the interaction detection module evaluated potential synergistic effect of coupling development of urbanization and ES when two factors act together. The specific formula is expressed as (62):


(12) $$\begin{array}{l} q=1-\frac{\sum_{h=1}^{L}N_{h}\sigma_{h}^{2}}{N\sigma^{2}} \end{array}$$

Where, *q* is the explanatory power of the impact factor, and the closer its value is to 1, the greater the explanatory power; *h* and *L* are stratification and stratification number of variable Y or independent variable X, respectively; *N*_*h*_ and *N* are the number of county units in layer *h* and the study area, respectively, $\sigma_{h}^{2}$ and σ^2^ are the variances of layer *h* and the whole area *Y*, respectively.

**Table 3:** Influencing factors of the CCD between urbanization and ESV in border counties.

**Category**	**Factor code**	**Factor**	**Unit**	**Indicator description**	**Indicator attribute**
Nature	X_1_	Precipitation	mm	Reflects regional rainfall abundance	+
	X_2_	Temperature	°C	Reflects regional thermal conditions	+
	X_3_	Average elevation	m	Measures regional topographic elevation	+
	X_4_	Soil organic carbon content	%	Reflects regional soil nutrient status	+
	X_5_	Net primary productivity (NPP)	gc/m^2^	Reflects surface vegetation production	+
	X_6_	Shannon diversity index	%	Reflects ecological landscape spatial distribution equilibrium	+
Economic	X_7_	Farmers' net income	Yuan	Reflects the living standards of rural residents	+
	X_8_	Tourist income	10^4^ yuan	Reflects tourism development level	+
	X_9_	Bilateral trade volume	$	Reflects cross-border trade development	+
	X_10_	Gross industrial output value above scale	10^4^ yuan	Reflects regional industrialization level	+
	X_11_	Traffic accessibility	km/km^2^	Reflects regional connectivity (ratio of railway/highway length to administrative area)	+
Social	X_12_	Population density	Person/m^2^	Reflects the density of regional population	+
	X_13_	Proportion of ethnic minority population	%	Measures regional sociocultural integration	+
	X_14_	Landscape fragmentation index	*n*/km^2^	Reflects landscape heterogeneity and fragmentation	–
	X_15_	Stable ecological land area	km^2^	Reflects regional environmental management (and area for ensuring the stable ES provision)	+
	X_16_	Government action	%	Proportion of local government expenditure in GDP	+

## 3 Result

### 3.1 Spatiotemporal variation of ESV and urbanization

**Figure 3:**
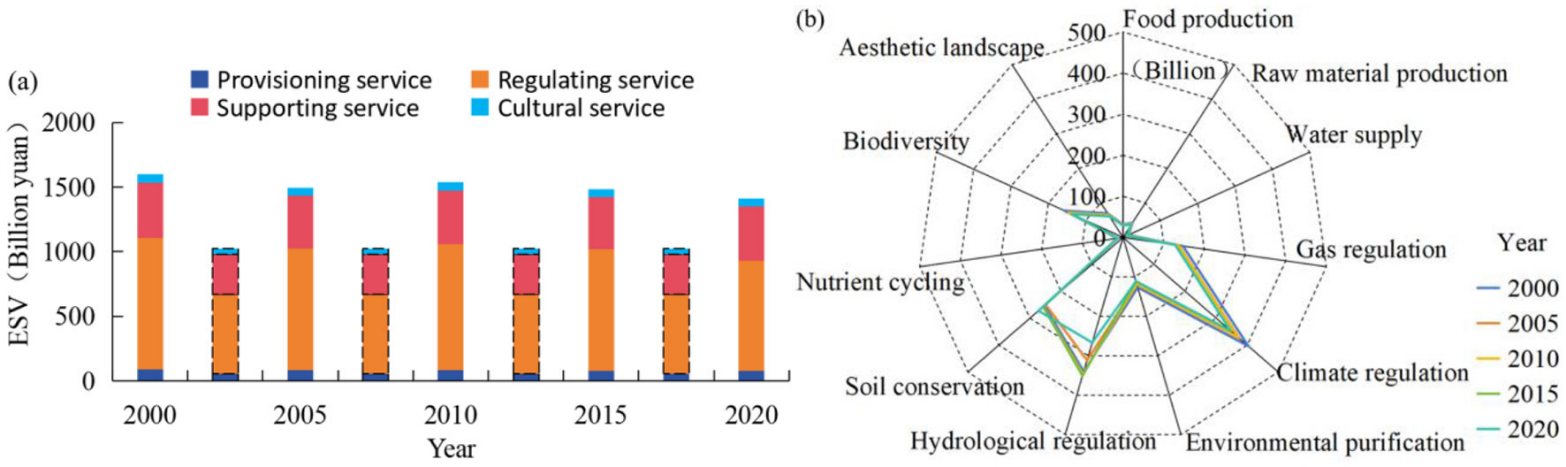
Overall change characteristics of ESV in border counties, 2000–2020.

The sensitivity analysis results of ESV and urbanization level show that the corresponding sensitivity coefficients were all < 1 from 2020 to 2020, indicating that ESV and urbanization are inelastic to their coefficients, and the research results are credible. During the study period, the ESV of China's western border counties exhibited a fluctuating decline (Figure 3a), decreasing from 16.01 × 10^2^ billion yuan (2000) to 14.11 × 10^2^ billion yuan (2020), representing an 11.88% reduction. The value of stable ESV was 10.22 × 10^2^ billion yuan. In terms of various kinds of ESVs and their variation (Figure 3b), the service value of maintaining nutrient cycling and water supply is the lowest, with annual mean values of 64.24 billion yuan and 13.41 billion yuan, respectively. While soil conservation values increased by 7.67%, all other ES categories experienced varying degrees of decline during the study period.

**Figure 4:**
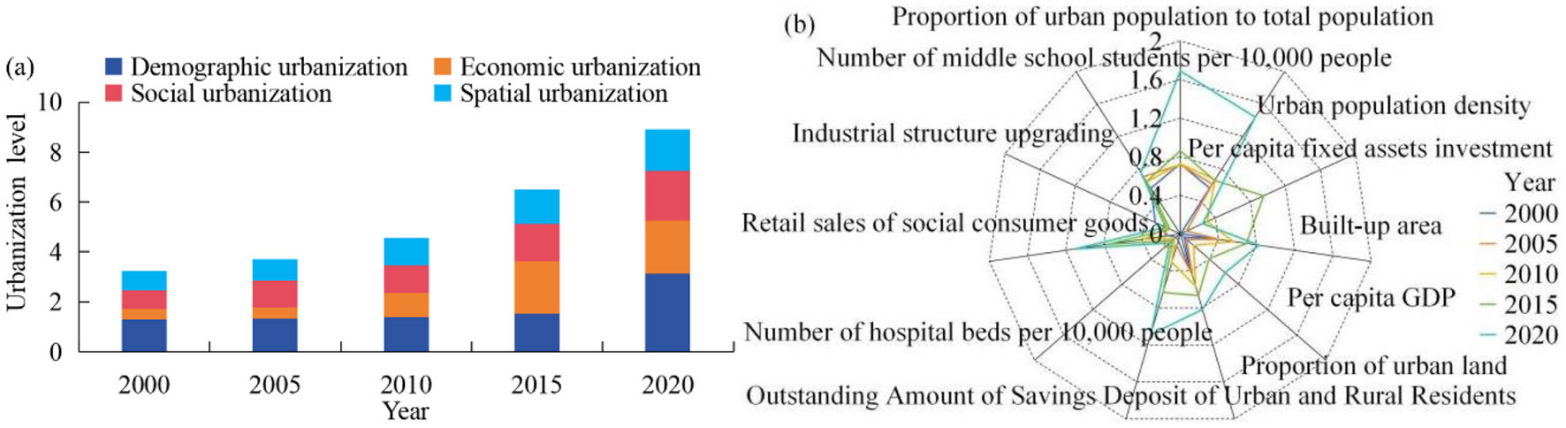
Overall change characteristics of urbanization in border counties, 2000–2020.

Different from the trend of ESV, the comprehensive urbanization level of the study area shows a gradient increasing trend (Figure 4), rising from 3.25 in 2000 to 8.91 in 2020, an increase of 5.66. Meanwhile, all kinds of urbanization levels showed a fluctuatings growth trend.

**Figure 5:**
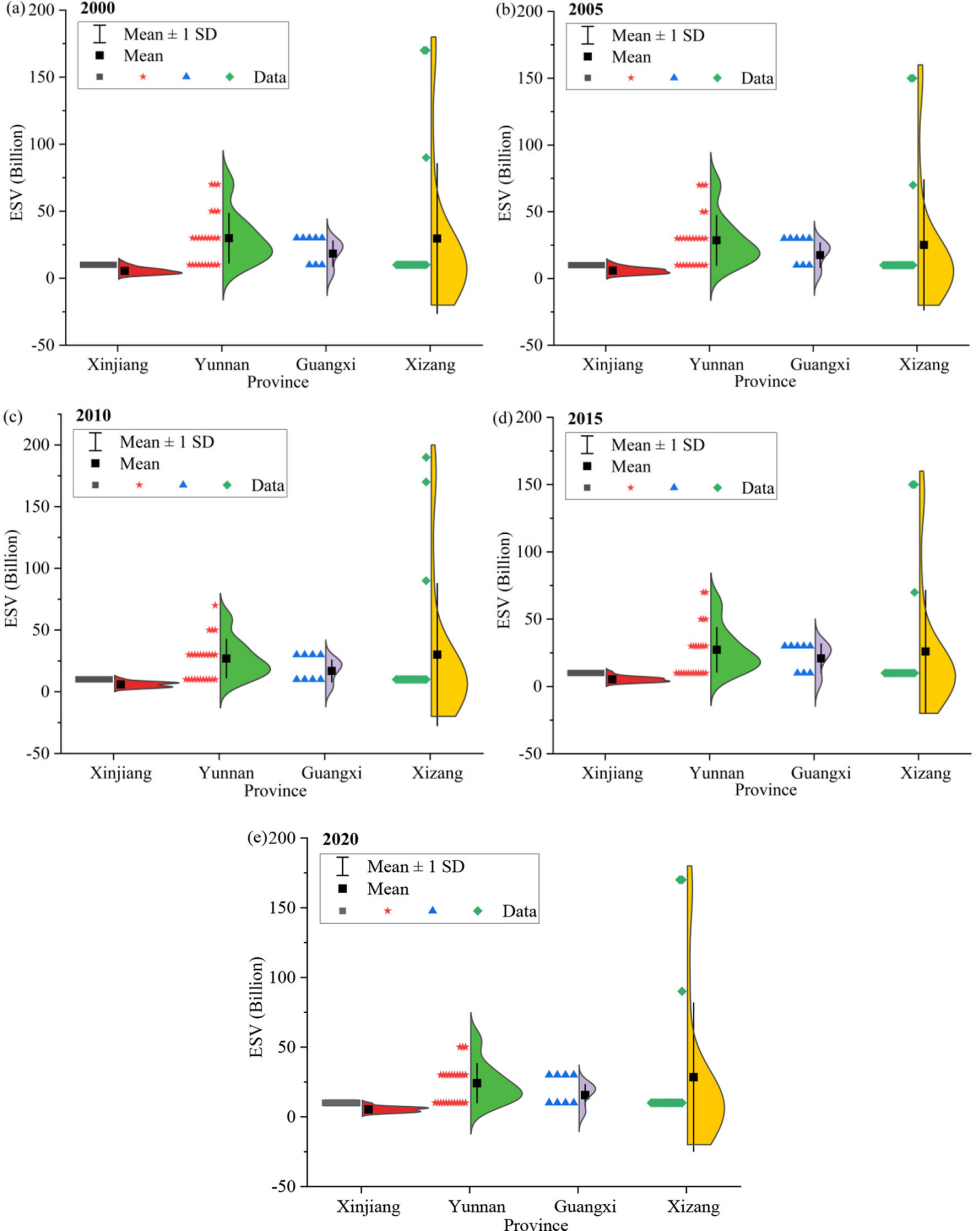
ESV variations in border counties, 2000–2020.

The border counties in the study area are managed by four provincial administrative units: Xinjiang, Yunan, Guangxi, and Xizang. Therefore, we conducted analyses from county and provincial perspectives, respectively (Figure 5). From 2000 to 2020, ESV declined gradually in 80% of the border counties in the study area. In particular, Mengla County and Jinghong City had the largest ESV decreases, with reductions of 21.01 billion yuan and 16.69 billion yuan, respectively, both located in Yunnan Province. The border counties with the highest and lowest ESVs are in Xizang, with annual mean values of 162.93 billion yuan and 1.05 billion yuan, respectively.

**Figure 6:**
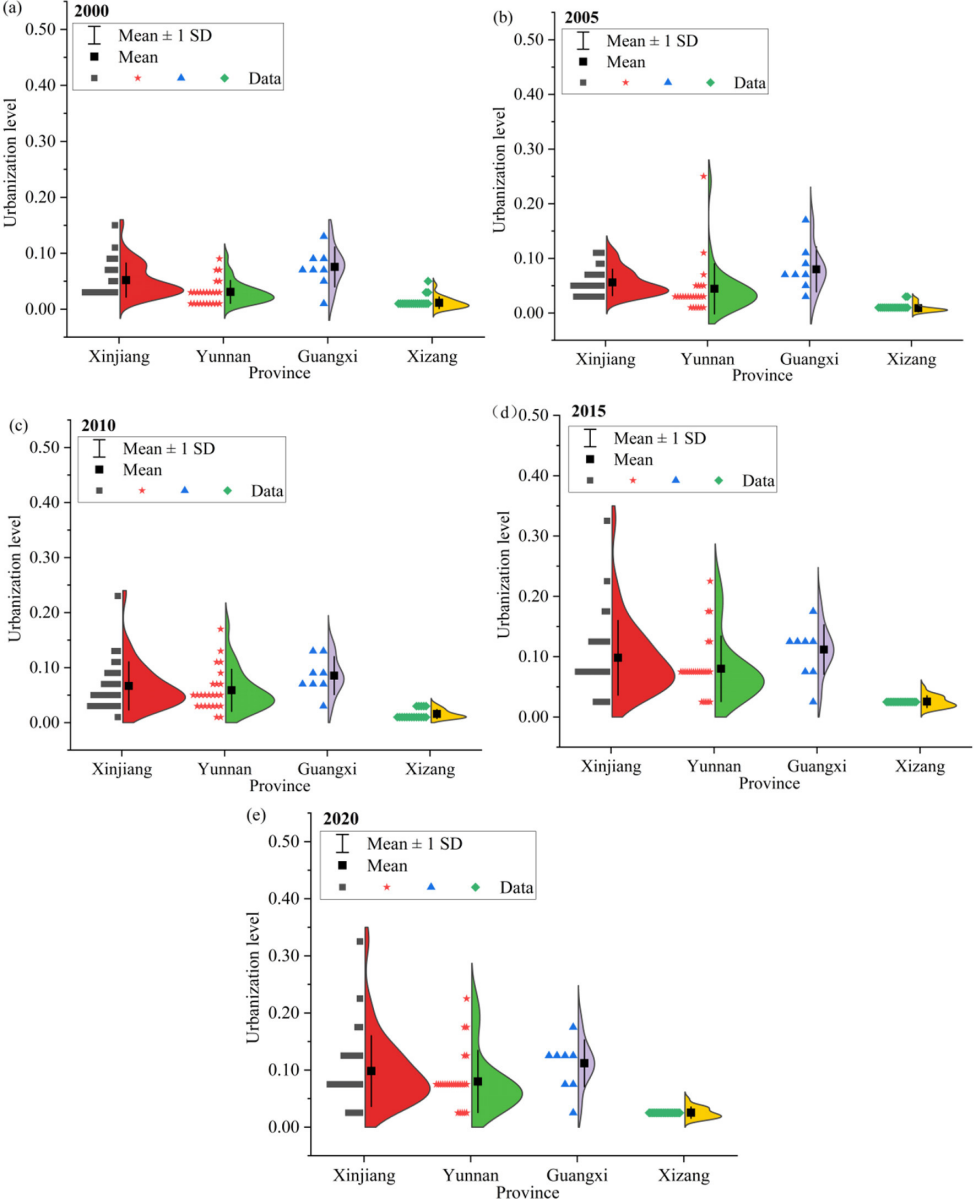
Urbanization variations in border counties, 2000–2020.

From 2000 to 2020, urbanization in border counties increased annually, but significant disparities emerged and widened over time (Figure 6). During this period, the urbanization of border counties in Guangxi was the highest, and that of Xizang was the lowest, with annual mean values of 0.11 and 0.02, respectively. As of 2020, the border counties with the highest and lowest urbanizations are Hami City in Xinjiang and Gar County in Xinjiang, respectively, reaching 0.36 and 0.04, respectively. Within-province disparities of the urbanization were also pronounced: Guangxi exhibited the largest gap (0.32), while Xizang had the smallest (0.03).

**Figure 7:**
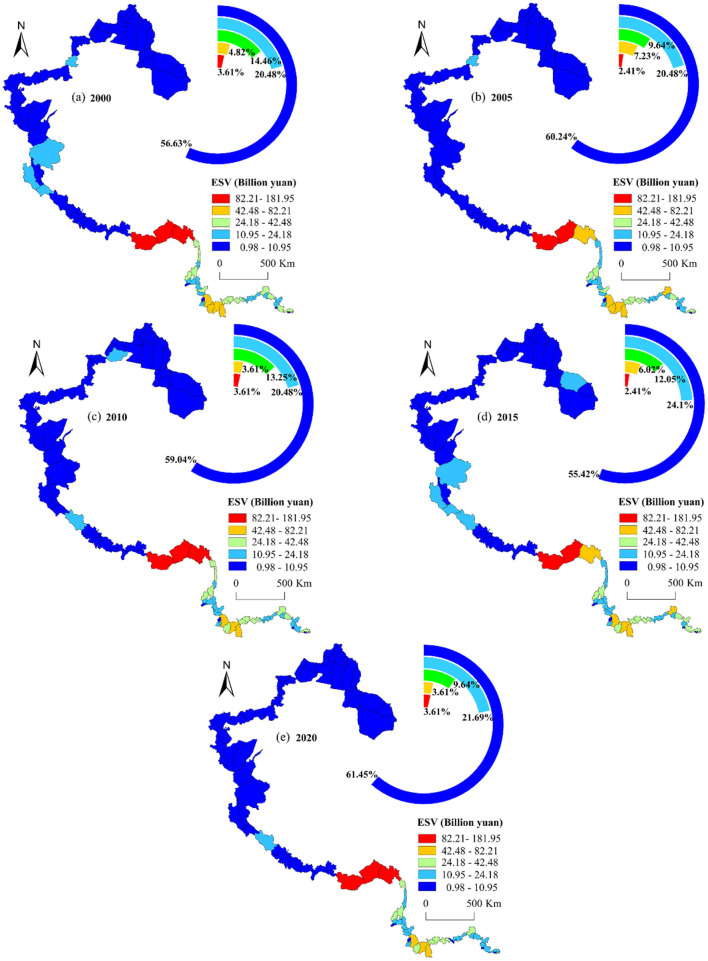
Spatial conversion of ESV in border counties, 2000–2020.

The ESV and urbanization levels of China's western border counties were classified into five categories from high to low using natural breakpoint method in ArcGIS. According to the results (Figure 7): from 2000 to 2020, ESV in China's western border counties generally exhibited a spatial pattern of high values in the south and low values in the north. In particular, regions with higher ESV were mainly concentrated in southern Xizang and Yunnan, while lower ESV regions primarily located in northern part of Xinjiang and Xizang. During the study period, more than 55% of border counties had ESV values ranging 0.98–10.95 billion yuan) with the number of counties in this range showing a fluctuating increase. The counties with ESV exceeding 82.21 billion yuan included Cuona, Zayu, and Medog County in southern Xizang.

**Figure 8:**
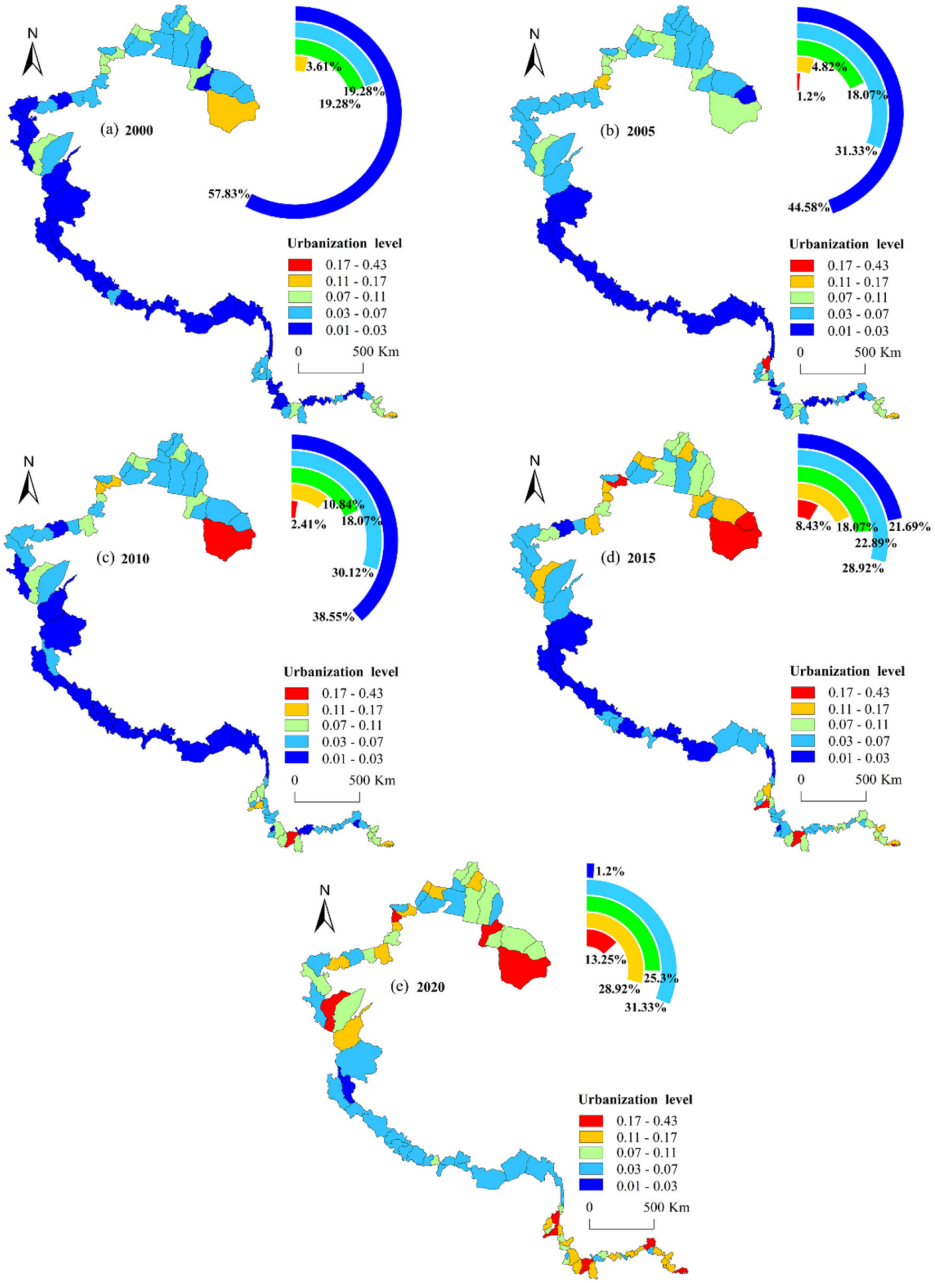
Spatial conversion of urbanization in border counties, 2000–2020.

The urbanization levels in the study area exhibited significant spatial heterogeneity (Figure 8): characterized by a multi-center pattern with, high values at both ends and low values in the center. From 2000 to 2020, the proportion of border counties with low urbanization (0.01–0.03) declined sharply, from 57.83% to 1.2%. These counties were primarily concentrated in southern Xinjiang and Xizang. Conversely, the number of counties with urbanization above 0.17 increased year by year, rising from 0% in 2000 to 13.25% in 2020. In terms of space, this trend was observed in other three provinces except Xizang. The urbanization development shifted from a single-center to multi-center pattern, ultimately forming a linked development network in the southern region.

### 3.2 Spatiotemporal variation of CCD between ESV and urbanization

#### 3.2.1 Characteristics of temporal variation

**Figure 9:**
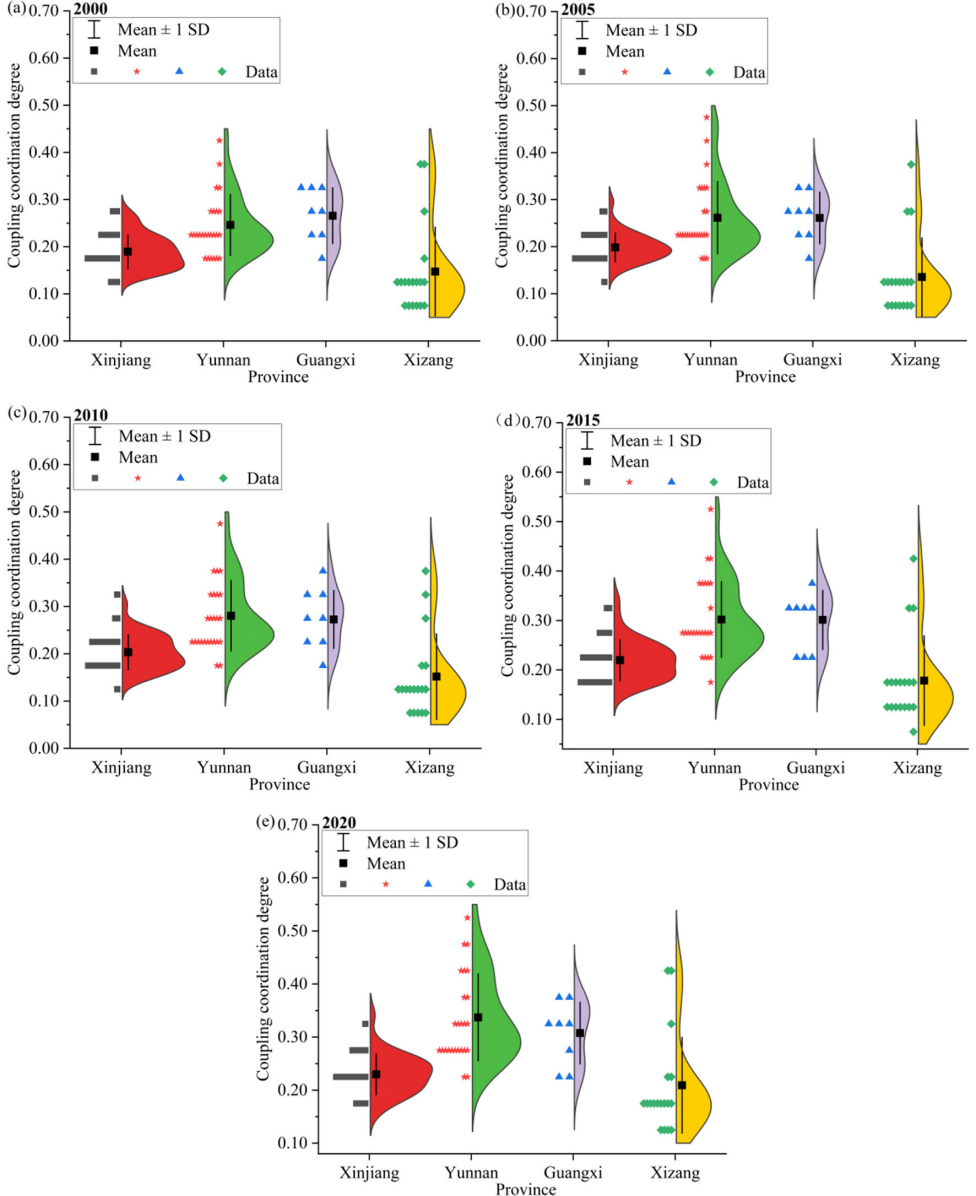
CCD variations in border counties, 2000–2020.

The spatiotemporal distribution patterns of ESV and urbanization in China's western border counties exhibit distinct differences and their coupling coordination degree (CCD) also reflects significant spatiotemporal variations (Figure 9). From 2000 to 2020, CCD increased across all border counties except Zhaosu County, which experienced a slight decline of 0.01. Among the counties, Jinghong City and Tengchong County in Yunnan Province had the highest CCD, with annual mean values of 0.41 and 0.49, respectively. In contrast, the lowest CCD values were primarily observed in Xizang. In addition, the CCD in the border counties of different provinces also shows considerable differences. The annual mean CCD values of border counties in Xinjiang, Yunnan, Guangxi, and Xizang all exhibited an increasing trend from 2000 to 2020. In particular, the mean value of CCD values of border counties in Yunnan and Guangxi were the highest, at 0.29 and 0.28, respectively. In contrast, Xinjiang and Yunnan had the lowest values, at 0.21 and 0.16, respectively. The mean CCD values of the border counties in Yunnan and Xizang increased the most, by 0.09 and 0.06, respectively.

#### 3.2.2 Characteristics of spatial pattern variation

**Figure 10:**
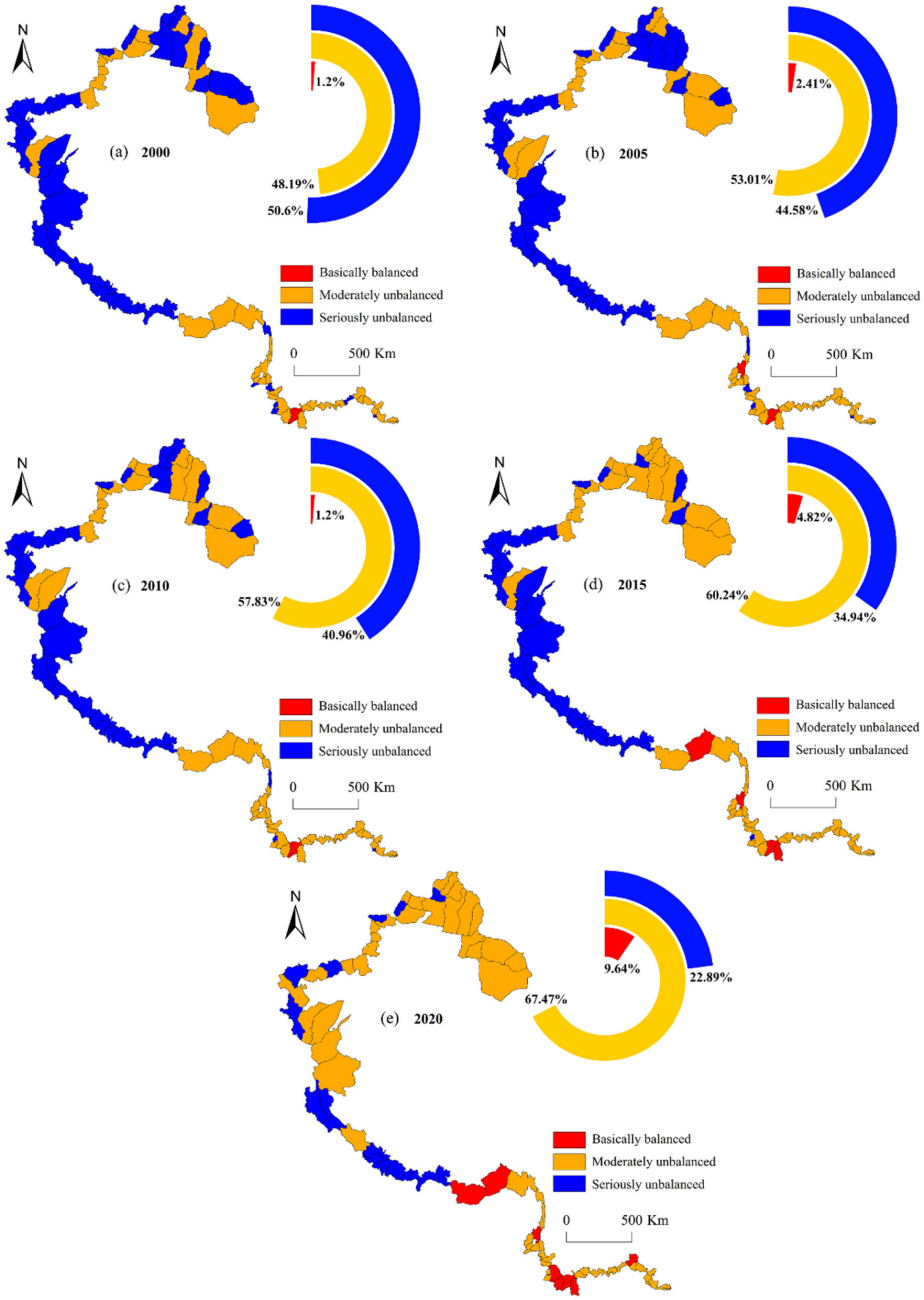
Spatial distribution pattern of CCD in border counties, 2000–2020.

The CCD between ESV and urbanization exhibits a distinct north-south spatial gradient, with higher values concentrated in southern regions and lower values prevalent in northern areas (Figure 10). From 2000 to 2020, the high-value areas were mainly distributed in southern Xizang and Yunnan, while the low-value areas were located in northern Xizang and most of Xinjiang. To be specific, the counties with seriously unbalanced CCD were mainly distributed in Xinjiang and Xizang, with their proportions reaching 22.89% in 2020 after declining year by year, a decrease of 27.71%. Counties with basically balanced CCD are mainly in Yunnan and southern Xizang, with their proportion rising from 1.2% in 2000 to 9.64% in 2020, an increase of 8.44%. The counties with moderately unbalanced CCD are dispersive and tend to expand toward the center from the southern and northern ends of the study area. Their proportion increases to 60.24% in 2020, rising by 12.05%. In addition, the CCD of border counties in Xinjiang and Yunnan increased significantly.

#### 3.2.3 Coupling coordination type

**Figure 11:**
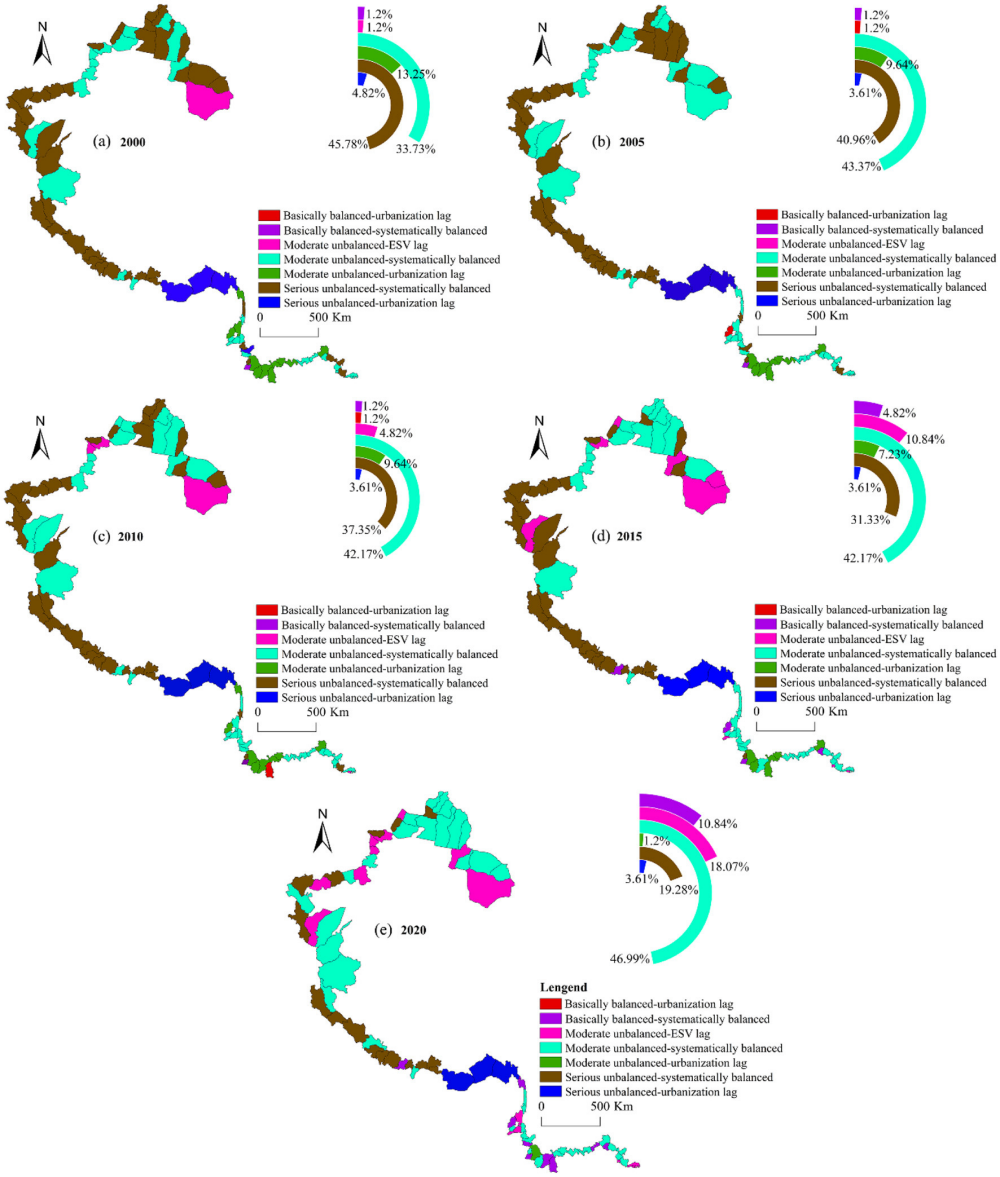
CCD types of border counties, 2000–2020.

Further analysis was conducted on the type of coupling coordination between ESV and urbanization in China's western border counties (Figure 11). Over the period from 2000 to 2020, the majority of counties within the study area exhibited coupling coordination that were either moderately unbalanced-systematically balanced or seriously unbalanced-systematically balanced, with respective annual average proportions of 41.69% and 34.94%. Notably, the proportion of counties characterized by moderately unbalanced-systematically balanced coupling coordination increased steadily, reaching 46.99% by 2020. In addition, the number of counties with ESV lagging increased over time, and predominantly located in Xinjiang. Conversely, the counties with urbanization lag were mainly situated in the southern regions of Yunnan and Xizang. Further investigation revealed that the counties with urbanization lag in the study area could be categorized into two types: seriously unbalanced-urbanization lag and moderately unbalanced-urbanization lag. Among these, the number of border counties with seriously unbalanced-urbanization lag was relatively small, accounting for an annual average proportion of 3.85%, and these counties were predominantly located in the southern part of Xizang. Additionally, the counties with moderately imbalanced -urbanization lag coupling experienced a shift from urbanization lag to system balance. The proportion of such counties decreased rapidly from 13.25% in 2000 to 1.2% in 2020, with the majority of these counties located within Yunnan Province.

The CCD model is capable of analyzing the relationship between ESV and urbanization but is not designed to determine whether the two systems are moving in the same direction. To ascertain whether the ES and urbanization are progressing synchronously, this study further categorized the types of coupling development between the two systems into four categories: ESV and urbanization grow synchronously (ER-UR); ESV increases while urbanization declines (ER-UD); ESV declines while urbanization increases (ED-UR); and ESV and urbanization decline synchronously (ED-UD).

**Figure 12:**
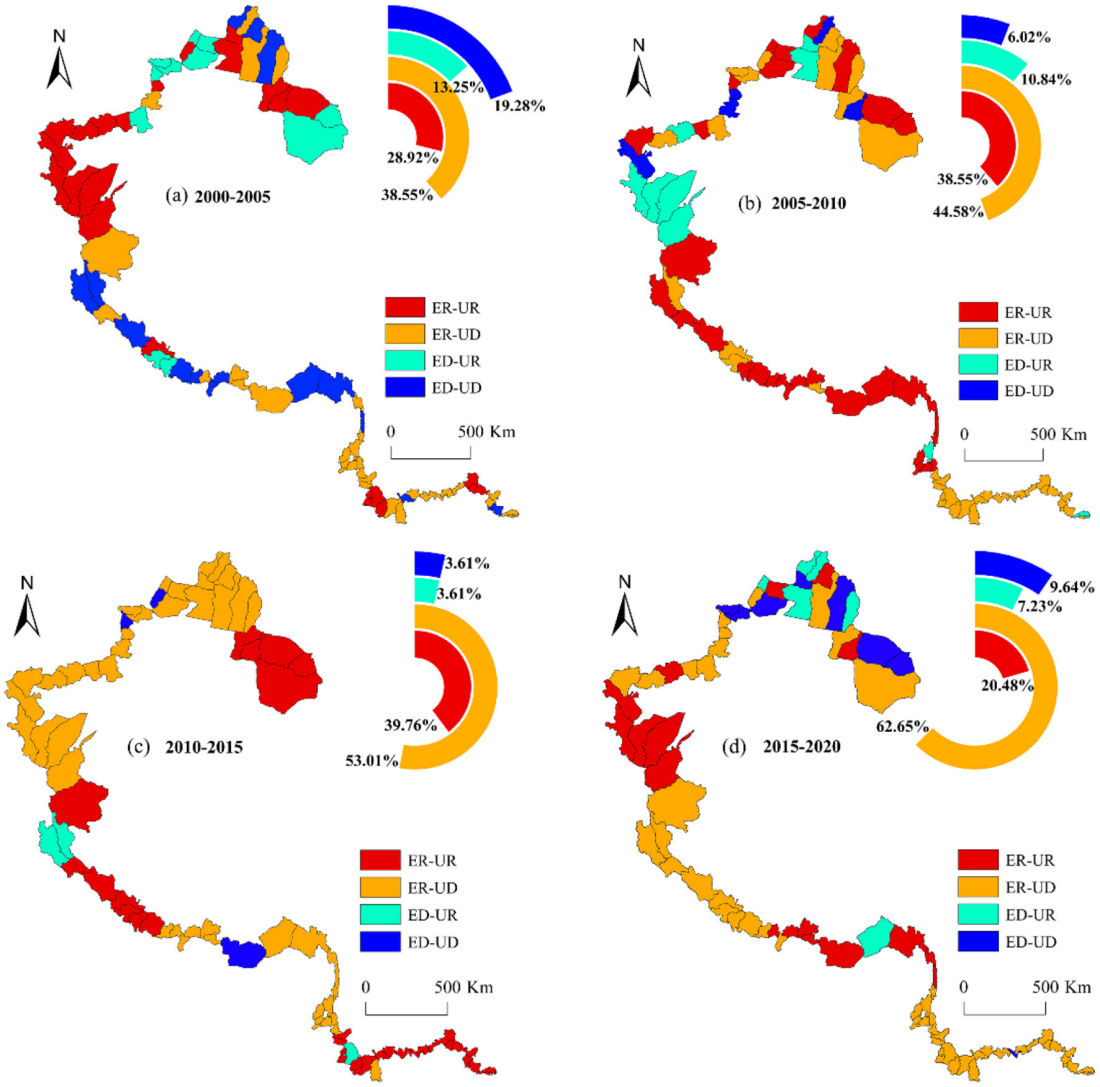
Coupling coordination types of ESV and urbanization in border counties, 2000–2020.

From 2000 to 2020 (Figure 12), the coupling development types in China's western border counties were predominantly ER-UD and ER-UR, with the annual mean proportions of 49.70% and 31.93%, respectively. From temporal perspective, the number of counties with ER-UR fluctuated and decreased to 20.48% in 2020, representing a decline of 18.07%. In contrast, the number of counties with ER-UD increased steadily, with the proportion increasing from 38.55% in 2000 to 20.48% in 2020. The number of border counties with ED-UD and ED-UR initially decreased and then increased with proportions of 9.64% and 7.23% in 2020, respectively. These results show that most border counties in the study area struggle to achieve a balance between ecological protection and urbanization development. Moreover, some counties experienced a simultaneous decline in both ecological protection and urbanization levels.

From a spatial perspective, counties characterized by the ED-UD coupling development type have transitioned from a pattern of overall dispersion to one of local agglomeration. In recent years, these counties have been predominantly located in the northern region of Xinjiang. Currently, the pastoral areas in northern Xinjiang are undergoing a significant transition from traditional to modern practices. This shift has inevitably led to irrational overstocking of livestock, thereby intensifying the pressure on grassland ecosystems. Additionally, the implementation of grassland ecological conservation policies has constrained the income growth of local herders while simultaneously slowing the pace of urbanization in these regions. In recent years, the majority of border counties that were previously characterized by the ER-UR coupling development type have shifted to the ER-UD type. This trend underscores the necessity for an in-depth discussion on how to achieve the coordinated development between ES and urbanization in border counties.

### 3.3 Determination of dominant impacting factors

#### 3.3.1 Dynamic obstacle factors of the CCD

**Figure 13:**
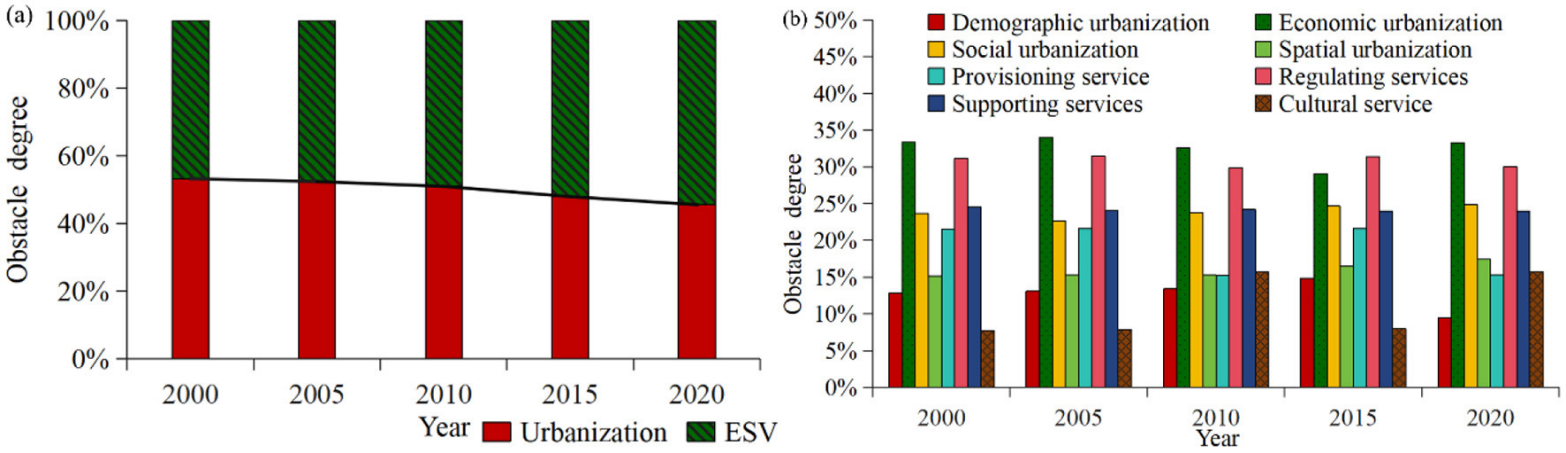
Obstacle degrees of systems and subsystems in urbanization and ESV, 2000–2020.

The obstacle degree model was employed to calculate the obstacle degrees of each subsystem and indicator within the coupled system. This approach enabled the identification and analysis of the main obstacle factors, thereby exploring the main obstacle factors impacting on the CCD of ESV and urbanization in border counties (Figure 13). From 2000 to 2020, the obstacle degree of urbanization system was marginally higher than that of ES system, with annual mean obstacle degree of 50.01% and 49.99%, respectively. However, the fluctuation trends of the obstacle degrees of both systems varied. To be specific, the obstacle degree of the urbanization system decreased annually from 53.23% in 2000 to 45.55% in 2020, while that of ES system increased in wave, reaching 54.45% in 2020. This indicates that ESV and urbanization have similar effects on the CCD of ESV and urbanization in the study area. As natural or human disturbances impact the ecosystem and lead to a continuous decline in the ESV, its constraining effect on the CCD of ESV and urbanization gradually intensifies.

From 2000 to 2020, the differences in the sub-systems and indicators of the urbanization and ESV system were substantial, with distinct development trends over time. Generally, the obstacle degrees of economic urbanization and regulating services were the highest, while those of cultural services and demographic urbanization were the lowest. Concretely, the obstacle degree of each subsystem within the urbanization system were characterized by the following order: economic urbanization > social urbanization > spatial urbanization > demographic urbanization, with annual mean obstacle degree of 32.46%, 23.91%, 15.91%, and 12.72%, respectively. From the temporal trend of the obstacle degree, the obstacle degree of each subsystem remains relatively stable over time. Economic and social urbanization factors play a decisive role, which is the key to balance the development of ESV and urbanization. In the ESV system, the annual mean obstacle degrees of the subsystems are orders as follows: regulating service (30.79%) > supporting service (24.16%) > provisioning service (19.07%) > cultural service (10.98%). With time passing by, the obstacle degree of the cultural service subsystem increased by 7.96%, while the other subsystems exhibited minimal change. This indicates that the weakening of ecosystem cultural service capacity in the study area is progressively reinforcing the obstacles to the CCD.

**Figure 14:**
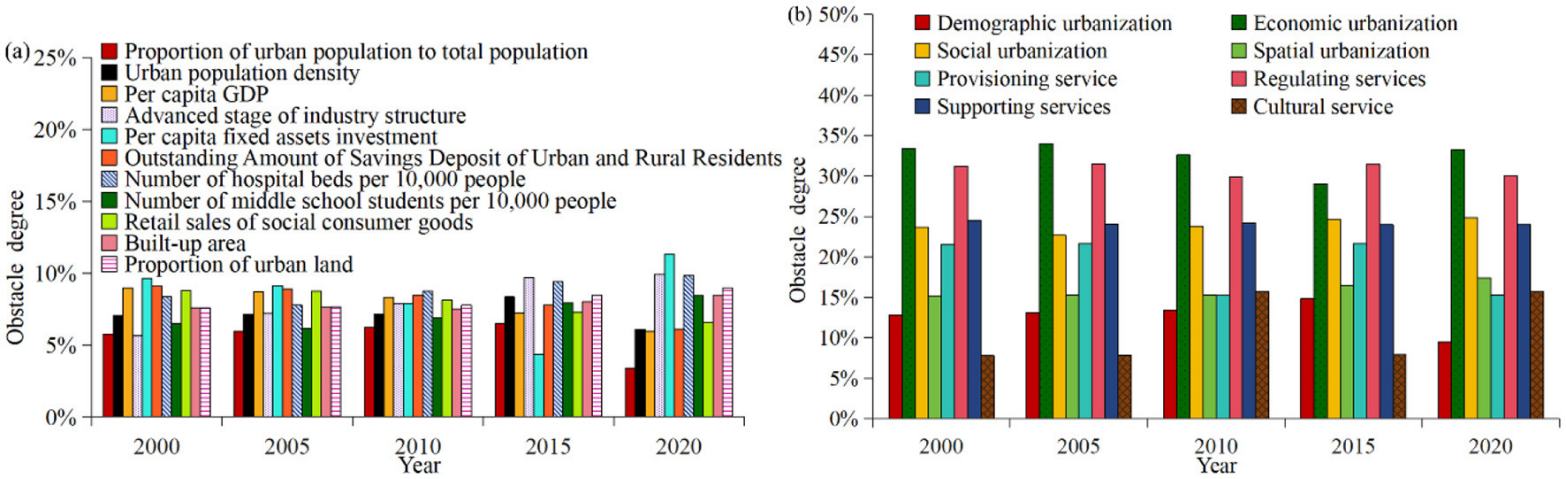
Obstacle degrees of various indicators in urbanization and ESV, 2000–2020.

As illustrated in Figure 14 from 2000 to 2020, the obstacle degrees of the number of per capita hospital beds and the per capita fixed assets investment within the urbanization system were the largest, with an annual mean obstacle degree of 8.83% and 8.47%, respectively. Conversely, the obstacle degrees of urban population advancement and urban population size were the lowest, with an annual mean obstacle degrees of 5.57% and 7.14%, respectively. These findings indicate that deficiencies in social protection and industrial development power are the primary factors impeding the coupling development of ESV and urbanization. In addition, the obstacle degrees of the advanced stage of industrial structure, the number of middle school students, per capita fixed assets investment, and the number of hospital beds exhibited the most significant increases rising by 4.27%, 1.91%, 1.67%, and 1.47%, respectively. This further indicates that the enhancing social protection, promoting the transformation of regional industrial structure, and boosting industrial development vitality can significantly contribute to the coupling coordinated development of urbanization and ESV in the study area.

During the study period, within the ESV system, soil conservation and climate regulation had the highest obstacle degrees, with annual mean obstacle degree of 8.50% and 7.92%, respectively. In contrast, food production and water supply had the lowest obstacle degree, with annual mean obstacle degree of 7.42% and 6.29%, respectively. Regarding the trends in obstacle degrees, all indicators except soil retention and food production experienced increases, with the most significant rises observed in hydrological regulation and climate regulation, which increased by 0.42% and 0.16%, respectively. These results suggest that, beyond soil erosion prevention, improving the regulatory capacity of ecosystem services is crucial for facilitating the coordinated development of ESV and urbanization in the study area, warranting further attention.

#### 3.3.2 Dynamic driving factors of the CCD

**Figure 15:**
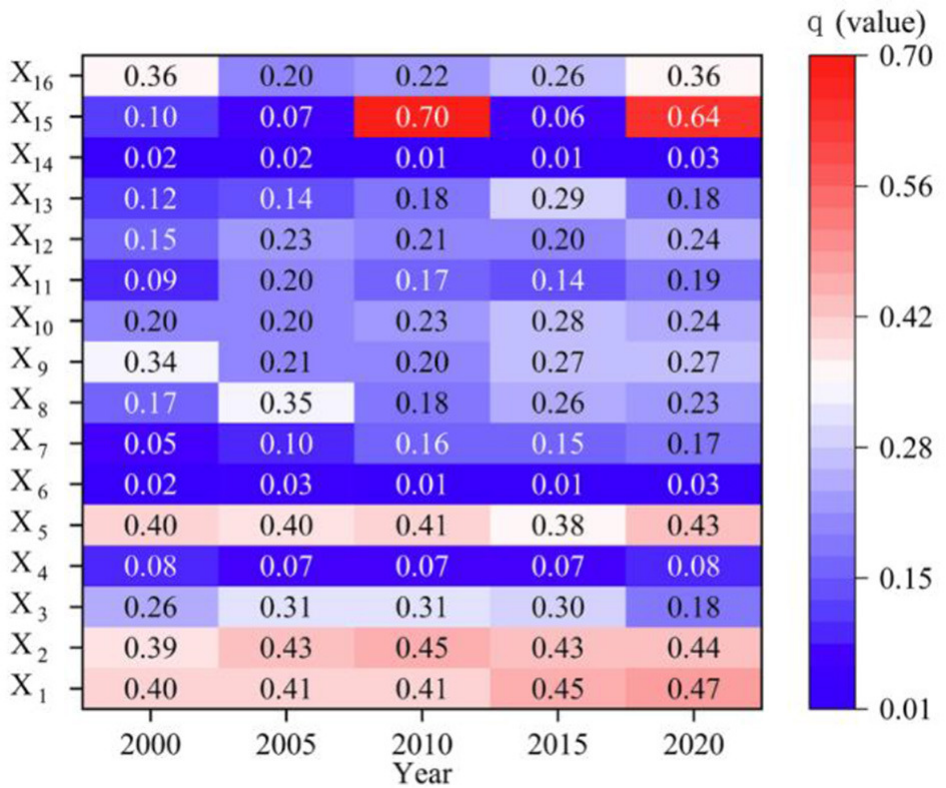
Explanatory power of driving factors for CCD, 2000–2020.

The factor detector of the geographic detector was employed to calculate the influence of 16 external factors, including natural factors and human activities, on the CCD in China's western border counties (Figure 15). From 2000 to 2020, the primary factors impacting on the CCD in these counties were *X*_1_ (annual mean rainfall), *X*_2_ (annual mean temperature), *X*_5_ (NDVI), *X*_15_ (stable ecological land area), and *X*_16_ (government action), with the mean *q* values of 0.43, 0.43, 0.40, 0.31, and 0.28, respectively. It should be noted that the explanatory power *q* of each impacting factor has passed the significance test at *P* < 0.01, indicating statistical significance. From a multidimensional analysis, it was found that natural factors such as *X*_1_ (annual mean rainfall), *X*_2_ (annual mean temperature), and X_5_ (NDVI) have the strongest explanatory power for the CCD. Among economic factors, *X*_9_ (bilateral trade volume), and *X*_8_ (tourist income) have the highest explanatory power. Social factors, including *X*_15_ (stable ecological land area) and *X*_16_ (government action), also exhibit strong explanatory power. In recent years, the driving role of *X*_15_ (stable ecological land area) has become more pronounced, playing a dominant role with a *q* value of 0.64 in 2020.

**Figure 16:**
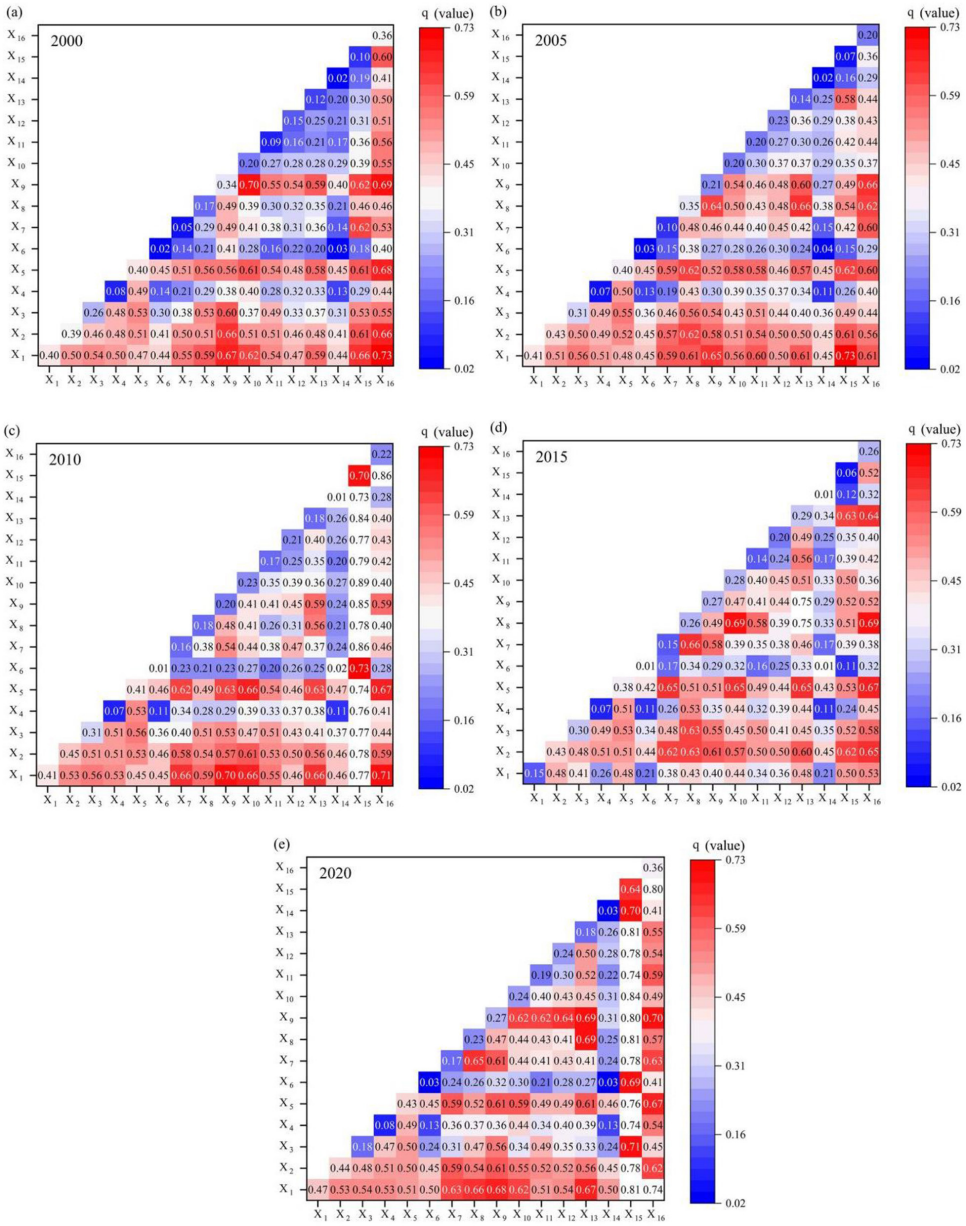
Interaction detection of impacting factors on CCD, 2000–2020.

The interaction detector within the geographic detector is capable of identifying the interactions between different factors and evaluating the strength of the interaction of two factors relative to that of a single factor (63). This involves assessing whether the combined effect of two factors enhances or diminishes the coupling effect between the ESV and urbanization. Therefore, after conducting an interactive detection analysis on the aforementioned 16 impact factors, the following conclusions were drawn (Figure 16): The influence of the interaction of two factors on the CCD is generally higher than that of a single factor. From 2000 to 2020, the factor *X*_15_ (stable ecological land area) and *X*_16_ (government action) predominantly impacted the interaction with other factors. Additionally, *X*_8_ (tourist income) exhibited significant interaction with *X*_13_ (minority population proportion) and *X*_15_ (stable ecological land area). The factor *X*_3_ (elevation) also showed notable interaction with *X*_9_ (bilateral trade volume), *X*_8_ (tourist income) and *X*_15_ (stable ecological land area). In recent years, the combination of *X*_8_ (tourist income) and *X*_15_ (stable ecological land area) has demonstrated the highest explanatory power. This finding underscores that increasing the stable ecological land area through the development of ecological protection zones in border counties can not only enhance ecological protection and functional restoration, but also foster the harmonious symbiosis of ESV and urbanization in border counties in combination with tourism development.

## 4 Discussions

### 4.1 Interaction between ESV and urbanization

The coupling coordinated development of ESV and urbanization requires increased attention to mitigate the potential for urbanization to severely degrade the ecological environment. This study, using China's western border counties as a typical case, investigates the spatiotemporal evolution of ESV, urbanization, and their coupling coordinated development over an extended period. The results indicate that as urbanization in these counties, ESV tends to decline, which is detrimental to regional sustainable development. Thus, promoting the coupling coordinated development of the ESV and urbanization is essential.

In terms of spatial distribution, ESV in border counties generally declines from south to north, consistent with the founding's of Gu et al. (64). This trend is primarily related to the regional natural endowments while the inter-annual variations are mainly impacted by climatic factors such as precipitation, temperature, as well as human activities (65). Border counties with high urbanization levels are scattered spatially, appearing in areas with both high and low ESVs. This indicates that the relationship between ES and urbanization is not completely opposite, but follows the relationship of CCD. The phenomenon described by Hu et al. (66), where ESV increases along with urbanization in the later stage of an inverted “U” development curve, is observed. Border counties with low urbanization levels are stably distributed in the central and northern parts of Xizang, the highest elevation area in the study region, indicating that high elevation hinders human activities and is an important factor impacting on urbanization (67). From these results, it can be concluded that the coupling development of ESV and urbanization in border counties is impacted by multiple factors, including both natural conditions and human activities. Therefore, it is of necessity to further explore the main factors impacting on CCD to guide the regulation of key factors impacting on CCD improvement, and effectively alleviate the conflict between urbanization development and ecological protection.

From 2000 to 2020, the CCD in China's western border counties has generally been low, far from achieving a highly balanced stage, thus indicating a substantial space for improvement (54). Most of the border counties in the study area gradually transitioned from a state of Serious-unbalanced-systematically balanced to either Moderate unbalanced-systematically balanced or Moderate unbalanced-ESV lag. Despite there has been some improvement in the CCD of these border counties, a significant conflict and potential crisis still exist between ESV and urbanization. In addition, an increasing number of border counties are experiencing either ESV increases coupled with urbanization declines or simultaneous declines in both ESV and urbanization, a trend that warrants full attention. To sum up, the majority of the border counties in the study area are confronted with the dual pressures of ecological protection and urbanization. Identifying the main factors impacting on CCD can provide objective grounds for formulating relevant policies and the development strategies for the study area and similar regions, thereby bringing practical significance to determining the priority issues that need to be managed (68).

### 4.2 Main factors impacting on CCD

On the basis of the existing studies, this study supplements and improves the factors impacting on the CCD of ESV and urbanization in China's border counties. First, the scope and sample size of the study were expanded. Hu et al. (66) focused on Yunnan Province's border counties to discuss the main driving factors affecting the CCD of ESV and urbanization in China's border counties. Considering China's extensive western border line and the regional differences among border counties, this study includes all counties along the western border. Additionally, while calculating ESV, the value equivalence factor was adjusted to account for the regional difference in the study area (21, 51). Furthermore, the study period was updated and extended to broaden the scope and enhance the representativeness of the results. Second, the impact factors of CCD were analyzed from a novel perspective. This study analyzes both the internal and external factors impacting the obstacle and driving factors of ESV and urbanization in China's western border counties, thereby providing more comprehensive and robust policy recommendations.

As shown by the results, there is little difference between ESV and urbanization systems in China's western border counties, both of which significantly impact on the CCD. Specifically, the obstacle degree of ESV increases over time, exerting a higher constraining effect on coupling coordination. From 2000 to 2020, the main obstacle factors in the urbanization system included the per capita number of hospital beds and per capita fixed assets investment. At the same time, the obstacle factors such as the advanced stage of industrial structure, the number of middle school students, the per capita fixed assets investment, and the number of hospital beds increase rapidly. This indicates that the main obstacles in urbanization system are mainly related to social protection and industrial structure adjustment. Addressing these obstacles promptly is crucial for promoting sustainable development in China's border regions (40). Soil conservation and climate regulation are the main obstacles to ESV. However, with time passing by, the obstacle degree of soil conservation function has declined significantly, while that of climate regulation and hydrological regulation functions has increased continuously. At the same time, various ESV in the border counties are imbalanced. The ESV of regulatory functions is much higher than the sum of the other functions, while the ESV of service functions is the lowest and continues to decline. These findings imply that, for ecological protection, it is essential not only to increase the protected area, but also to restore the regulatory function of the ecosystems, continuously strengthen the supply functions, and address the structural imbalance of ES functions (13, 34).

As for the driving factors impacting on CCD, natural factors (precipitation, temperature, NPP) have the largest driving effect on CCD in border counties, which is consistent with the conclusions of Hu et al. (66). In addition, the driving forces of stable ecological land area and government action are also substantial, with the explanatory power gradually increasing. This indicates that coordinated development between urbanization and ecosystem protection is significantly influenced by regional natural resource endowments. However, increasing stable ecological land area and enhancing government intervention capacity could effectively promote their coordinated development. To be specific, NPP and stable ecological land area can reflect the degree of ecological disturbance caused by human activities to some extent (69). NPP, stable ecological land area, and government action indicate that increasing the area of ecosystems with high vegetation coverage or high ESV, such as forests, wetlands and grasslands, under the government initiatives can alleviate ecological disturbances caused by human activities and effectively enhance the CCD in border counties (33, 68). During the study period, the interaction of two factors on CCD in border counties were found to be higher than those of single factors, suggesting that the improvement of CCD in border counties should consider the coordination of multiple driving mechanisms. The interaction between tourist income, minority population proportion, and stable ecological land area has significant explanatory power, indicating that border counties can promote the development of local tourism by expanding stable ecological land area and leveraging minority culture, thereby improving the local CCD. Li et al. (67) identified elevation as an important factor impacting on CCD, which this study further confirms to have significant driving force in conjunction with bilateral trade volume, tourist income, and stable ecological land area. This implies that for border counties located in high elevation areas, increasing stable ecological land area and promoting tourism and border trade are the key practical directions to achieve the synchronous growth of ESV and urbanization.

### 4.3 Limitations and prospect

The outcomes of this study contribute certain practical references for improving CCD in China's western border counties. However, this study still needs further enrichment and discussion in future research. First, the study scope of this study is limited to China's most tortuous border region, which is also the most complex area with most neighboring countries. In the future, all border counties in China should be included in the study. Second, considering the principle of consistent data sources and limited data availability, the time span of the study could not be further expanded. Future research is still needed to supplement the findings. Third, the scale of this study is focused on county level; thus, discussions should be further expanded in the future to more refined scales or larger scales, such as the municipal and provincial levels.

Although our study has identified the key influencing factors and obstacle factors of the CCD in border counties and proposed corresponding development strategies, it has not conducted scenario-based simulations of CCD under coordination and constraints of policy regulation, environmental protection, regional cooperation development and tourism development. Future studies should focus on the multi-scenario prediction and simulation of CCD. Such efforts will enable precise anticipation of CCD trajectories in border areas, providing a scientific basis and decision-making references for governments to coordinate the planning of ecosystem protection and urbanization development.

### 4.4 Policy implications

Based on the findings of this study, the following policy implications are proposed to achieve the sustainable growth of ESV and urbanization in the western border counties and similar border counties of China:

(1) Enhancing ecological protection and restoration: For border counties with moderately unbalanced CCD characterized by ESV lag, it is imperative to delineate the ecological protection baselines to ensure a stable ecological area of a certain scale. This involves promoting the establishment of ecological protection zones to increase the stable ecological land area, emphasizing the restoration of ecosystem regulation functions, and safeguarding the authenticity and integrity of ecosystems. These measures can help maintain ecological stability and enhance the overall ESV in these regions.
(2) Strengthening social security and industrial development: For border counties with moderately unbalanced CCD characterized by urbanization lag, it is important to improve the level and scope of medical security and medical resource supply, intensify investment in educational resources, and meet the actual needs of border education. In addition, continuous optimization of industrial resources allocation, attention to industrial upgrading and transformation, and improvement of production efficiency and circulation coordination are essential to reduce resource waste and pollution emissions. These actions can improve the quality of life and economic development in these counties.
(3) Promoting tourism and border trade development. For border counties with seriously unbalanced CCD, exploring the local minority cultures of the minorities and leveraging the favorable ecological environment to activate the border tourism development can promote the growth of distinctive border tourism. Simultaneously, strengthening cooperation and exchanges with neighboring countries through cross-border tourism cooperation can enhance regional integration. Accelerating the construction of border ports, and formulating preferential policies for border trade can further promote border trade and foster new momentum for the opening up and development of border regions. These initiatives can help bridge the gap between ESV and urbanization, leading to more balanced and sustainable development.

## 5 Conclusions

This study selected China's western border counties as the study area, analyzed the evolution of ESV, urbanization and their CCD over a long time series. Meanwhile, the main obstacle factors and driving factors impacting on CCD were examined, and corresponding policy implications were developed based on the study results. The main conclusions are as follows:

(1) Distinct spatiotemporal disparities exist in ESV and urbanization across China's western border counties during the study period. Temporally, the level of urbanization in these border counties increased steadily, while ESV showed a fluctuating downward trend from 2000 to 2020. Spatially, ESV in border counties declined from south to north, while the urbanization level showed a multi-center distribution, with higher values at both ends of the study area and lower values in the middle. These findings indicate that the factors driving the coupling of ESV and urbanization are complex and require further exploration.
(2) From 2000 to 2020, the CCD between ESV and urbanization in border counties generally improved, although the overall level remained low, with dominant coupling types being moderately unbalanced and seriously unbalanced. Only a small number of border counties achieved synchronous growth in ESV and urbanization, while some counties experienced simultaneous decline in both. Identifying the primary influencing factors of CCD is urgently needed to foster the integrated development of the two systems.
(3) Precipitation, temperature, and NPP are the main driving factors of the CCD, with two-factor interactions exerting stronger influences than single factors. Among these, the interactions of tourism, stable ecological area, and government action with other factors have the most significant driving force on the CCD between the two systems.
(4) To enhance the CCD between ESV and urbanization in China's western border counties and similar regions, local governments need to strengthen the management and maintenance of ecological reserves, focusing on the functional restoration of ecosystem services. Concurrently, policies and financial support should be allocated to border counties to enhance their social security, optimize industrial structure, promote tourism, and expand trade with neighboring countries. These measures will contribute to the sustainable and coordinated development of ESV and urbanization in border regions.

## Data Availability

The datasets presented in this study can be found in online repositories. The names of the repository/repositories and accession number(s) can be found in the article/supplementary material.
